# Companionship during facility-based childbirth: results from a mixed-methods study with recently delivered women and providers in Kenya

**DOI:** 10.1186/s12884-018-1806-1

**Published:** 2018-05-10

**Authors:** Patience Afulani, Caroline Kusi, Leah Kirumbi, Dilys Walker

**Affiliations:** 1 Obstetrics, Gynecology and Reproductive Sciences, UCSF, San Francisco, USA; 2 Institute for Global Health Sciences, UCSF, San Francisco, USA; 3 Kenya Medical Research Institute, Nairobi, Kenya

**Keywords:** Continuous support during labor and delivery, Birth companionship, Woman-centered care, Respectful maternity care, Kenya

## Abstract

**Background:**

Research suggests that birth companionship, and in particular, continuous support during labor and delivery, can improve women’s childbirth experience and birth outcomes. Yet, little is known about the extent to which birth companionship is practiced, as well as women and providers’ perceptions of it in low-resource settings. This study aimed to assess the prevalence and determinants of birth companionship, and women and providers’ perceptions of it in health facilities in a rural County in Western Kenya.

**Methods:**

We used quantitative and qualitative data from 3 sources: surveys with 877 women, 8 focus group discussions with 58 women, and in-depth interviews with 49 maternity providers in the County. Eligible women were 15 to 49 years old and delivered in the 9 weeks preceding the study.

**Results:**

About 88% of women were accompanied by someone from their social network to the health facility during their childbirth, with 29% accompanied by a male partner. Sixty-seven percent were allowed continuous support during labor, but only 29% were allowed continuous support during delivery. Eighteen percent did not desire companionship during labor and 63% did not desire it during delivery. Literate, wealthy, and employed women, as well as women who delivered in health centers and did not experience birth complications, were more likely to be allowed continuous support during labor. Most women desired a companion during labor to attend to their needs. Reasons for not desiring companions included embarrassment and fear of gossip and abuse. Most providers recommended birth companionship, but stated that it is often not possible due to privacy concerns and other reasons mainly related to distrust of companions. Providers perceive companions’ roles more in terms of assisting them with non-clinical tasks than providing emotional support to women.

**Conclusion:**

Although many women desire birth companionship**,** their desires differ across the labor and delivery continuum, with most desiring companionship during labor but not at the time of delivery. Most, however, don’t get continuous support during labor and delivery. Interventions with women, companions, and providers, as well as structural and health system interventions, are needed to promote continuous support during labor and delivery.

**Electronic supplementary material:**

The online version of this article (10.1186/s12884-018-1806-1) contains supplementary material, which is available to authorized users.

## Background

Over the past decade, the promotion of facility based delivery has been at the forefront of international efforts to reduce maternal mortality. Between 2003 and 2013, the percentage of women who delivered in health facilities increased in every region of the world [1]. However, retention, equity, dignity, and quality of care along the continuum of maternal health care remain a challenge [2–4]. Women’s experiences during childbirth are a powerful determinant of use of maternal healthcare services [5]. The World Health Organization (WHO) Quality of Care Framework for maternal and newborn health outlines access to emotional and social support of women’s choice as core to the experience of care and to achieving positive person-centered health outcomes [6]. Thus, WHO recommends that every woman is offered the option to experience labor and childbirth with a companion of her choice [7].

Different terms have been used for this recommendation, including companion of choice at birth, labor companion, birth companion, emotional support during birth, social support during labor and delivery, supportive companionship, and continuous support for women during childbirth [8–13]. But the evidence supporting the recommendation emphasizes continuous support during childbirth [9, 10]. The definition of continuous support during childbirth differs across studies and settings and can start at any time between conception to postpartum period. Generally, however, it involves the continuous presence of a companion during labor and delivery [9, 10]. In this paper, we use the terms birth companionship to refer to the provision of any type of support by a lay companion at any time during the intrapartum period, and continuous support during labor and delivery (CSLD) to refer to the continuous presence of the companion during labor and delivery. CSLD is thus a subset of birth companionship since not all birth companionship is continuous.

Common elements of support provided by birth companions include, emotional support (e.g. continuous presence and giving reassurance and praise), informational support (e.g. giving information about labor progress and advice regarding coping techniques), comfort measures (e.g. comforting touch, massage, warm baths/showers, encouraging mobility, and promoting adequate fluid intake and output), advocacy (e.g. helping women articulate their wishes to providers), and instrumental support (providing assistance with needs) [9, 10, 14]. Support can be provided by different types of companions, including trained support persons such as doulas (non-health care professionals trained to provide emotional and physical support to women before, during and after childbirth) or by companions chosen from a woman’s social network [9, 15].

Birth companionship is associated with increased satisfaction with healthcare services [9, 16]. Some studies also suggest that women who have birth companions are less likely to experience mistreatment during childbirth [17, 18]. In addition, CSLD has been shown to impact pregnancy outcomes. A recent Cochrane review of CSLD found that women who received continuous labor support were more likely to have shorter labor and spontaneous vaginal deliveries (i.e. give birth vaginally without the use of instruments or to have a cesarean section). In addition, they were less likely to use pain medication while giving birth and their babies were less likely to have low five-minute Apgar scores [9]. The effect of CSLD differed, however, depending on the type of companion and the setting. They found that CSLD was most effective at reducing caesarean birth when the companion was present in a doula role, as well as in settings where epidural analgesia was not routinely available. The effect of CSLD on birth outcomes was also stronger in settings where women were not permitted to have companions during labor. The authors concluded that continuous support from a person who solely provides support, is not a member of the woman’s own network, is experienced in providing labor support, and has a modest amount of training (such as a doula), appears to have the greatest impact on outcomes. Nonetheless, compared to having no companion, support from a chosen family member or friend appeared to increase women’s satisfaction with their childbirth experience [9]. The authors noted that none of the studies included in their review were from low-income settings and, therefore, recommended that future studies explore how continuous support can be best provided in different contexts, particularly in low-income settings.

Birth companionship is increasingly being included in the maternal health guidelines of many countries, including in low- and middle-income countries in Sub-Saharan Africa (SSA). For example, the National Guidelines for Quality Obstetrics and Perinatal Care in Kenya recommends that during the first stage of labor, providers should encourage women to have their chosen companions with them and make arrangements to accommodate birth companions or male partners; and in the second stage of labor providers should “allow and encourage her birth companion/male partner to be present” [19]. Yet little is known about the extent to which this is actually practiced in Kenya, as well as in most of SSA. The research is equally limited on women’s perceptions and preferences for birth companions, and on providers’ perceptions of birth companions in these settings [11–13, 20, 21]. No study to our knowledge has comprehensively examined birth companionship in Kenya. Little is therefore known about the prevalence of birth companionship including CSLD, the types of companions used, women’s preferences for companionship, and the factors associated with these in Kenya. In contexts where women have little autonomy and their health seeking behavior is strongly influenced by sociocultural norms; and providers have greater autonomy, but operate with very limited resources, it is important to understand women’s preferences for companionship and providers perceptions about providing it, to guide efforts towards promoting birth companionship. The current study aims to fill this gap in evidence.

Furthermore, while most of the works on birth companionship have examined it as a single unit, we chose to make some distinctions based on our field experiences. The first distinction is between having a companion present and having continuous support. This is motivated by the fact that, while a woman might have a companion present, the support provided may not be continuous for various reasons. The second distinction is between labor support, delivery support, and post-delivery support. This distinction is to enable us to understand if women’s preferences for support differ at these different stages of the childbirth process. In addition, these distinctions are important because, when support is not continuous and is only provided at certain time points (like during labor but not delivery), the potential benefits may differ from those stated in CSLD literature. Our objectives, therefore, were to: (1) Assess the prevalence of birth companionship and CSLD in health facilities in a rural county in western Kenya; (2) Assess women’s desire for birth companionship; (3) Identify types of birth companions that women prefer; and (4) Examine factors associated with receiving CSLD, desire for companionship, and preference for male companions. In addition, we use qualitative data to examine: (1) women’s perceptions of birth companionship and their preferred companions; and (2) providers’ perceptions of birth companionship and barriers to ensuring CSLD for women.

## Methods

We employed mixed methods for this study. Mixed methods combine elements of qualitative and quantitative approaches to provide breadth and depth in our understanding of phenomenon [22]. Quantitative methods allow objective measurement of a particular reality, with a large enough sample to increase generalizability [23]. Quantitative methods are however limited in explaining the “why” of phenomena and how personal viewpoints, context and meaning produce multiple realities [23]. Qualitative methods help to address this weakness, but have limited generalizability because of practical limitations of sample size. Mixed methods were therefore appropriate to harness the strengths and counterbalance the weaknesses of quantitative and qualitative methods to provide a holistic understanding of birth companionship [23]. The quantitative methods provide data for measuring prevalence and examining statistical associations and the qualitative data help to explain the quantitative findings and provide rich descriptions of views and beliefs about birth companionship. In addition, we collected data from recently delivered women and maternity providers to obtain the complementary perspectives of both the recipients and providers of care.

### Setting

Data for this study were part of a larger study examining community perceptions of quality of maternity care in a rural county in western Kenya. The County has a population of about one million and an estimated 40,000 annual births [24]. Approximately 43% of the population live below the poverty line and only about 3% of women of reproductive age have more than a secondary education. About 24% of women of childbearing age are 15–19 years [25]. The total fertility rate of the county is 5.3 compared to the national average of 3.9. The County is one of 15 counties in Kenya that account for over 60% of maternal deaths in the country—with an estimated maternal mortality ratio of 673 deaths per 100,000 live births compared to the national average of 495 [26]. The number of nurses, clinical officers, and doctors, per 100,000 people in the county is 32, 19, and 4 respectively [27].

About 53% of births in the County occur in health facilities, compared to the national average of 61% [25]. Several factors account for low use of facility deliveries, including low perceived need of facility deliveries, poor physical and financial access to health services, and low perceived quality of care [28–30]. Additionally, sociocultural factors such as norms and values related to childbirth (e.g. beliefs about the causes of pregnancy complications) and women’s autonomy (e.g. women not being final decision makers on place of birth) interact with socioeconomic factors such as wealth and education to determine whether or not women seek health care during pregnancy or delivery [5, 31–33]. Thus, the low status of most childbearing women in the County (young, uneducated, poor, and unemployed) is likely a key determinant of the low use of services in the County. These factors also affect how women are treated in the health facility, which affects future health seeking behavior. Given evidence that birth companionship improves women’s birth experiences [5, 9, 17], understanding and respecting women’s desires and preferences for birth companions could positively impact health seeking behavior during childbirth in the County.

### Data sources

We used 3 data sources: surveys and focus group discussions with recently delivered women and in-depth interviews with providers. Eligible women were 15–49 years and delivered in the 9 weeks preceding the study. Eligible providers worked in a maternity unit in the county. Ethical approval for the study was provided by the authors’ institutions. All participants provided written informed consent after receiving information about the study and received 200 KES (~$2) for their participation.

#### Surveys with women

Survey data were collected between August and September 2016 with eligible women. A multistage sampling approach was used to select women. First, the County was divided into eight strata based on its 8 sub-counties. Next, ten health units were randomly selected from each strata. Eligible women were then identified and recruited from the selected health units. The sampling procedures are documented elsewhere [34]. Twelve data collectors were trained to conduct the interviews—in English, Swahili, and Luo in private spaces in health facilities or in respondents’ homes. Data were collected using the REDCap electronic data capture tools hosted at the University of California-San Francisco [35]. Approximately one thousand women were interviewed, with a response rate above 98%. We use data from women who delivered in a health facility (894) and had no missing data on relevant variables for this analysis (*N* = 857–877 for different outcomes).

#### Focus group discussions (FGDs) with women

Between October and November 2016*,* we conducted eight FGDs—one in each sub-county—with 58 eligible mothers. We chose a focus group design to obtain qualitative data from women because our field experience in the region suggested women were more willing to discuss their experiences in groups with their peers than with an individual interviewer not familiar to them. The FGD procedures are documented elsewhere [36]. We recruited respondents from one randomly selected health unit in each sub-county that was not included in the survey. Women who met the eligibility criteria were purposively selected from the selected health unit. Each focus group consisted of six to ten women and lasted about 90 min. Two female research assistants with college degrees were trained by the first author to moderate each group discussion; one led the discussion using a discussion guide, and the other took notes and managed audio recording. Women were asked, among other things, if they were allowed to have a companion present during labor and delivery and how they felt when they were allowed or disallowed a companion. They were also asked if they desired birth companionship and why they desired or did not desire it. The discussions were conducted in Swahili or Luo, in private spaces in the community or health facility. Discussions were audio recorded, and simultaneously translated and transcribed.

#### In-depth interviews with providers

We conducted 49 interviews with maternity providers from 18 facilities across all the 8 sub-counties, which were selected for an intrapartum quality improvement project based on their relatively higher volume of deliveries. With permission from the county and facility heads to conduct the study, the research assistants approached maternity providers in selected facilities, briefed them about the study, and invited them to participate in the interviews. The interviewers used an interview guide with both closed and open-ended questions. Providers were asked, among other things, if women were allowed CSLD, their perceptions of CSLD, and barriers to providing it in their facilities. Interviews were conducted in English, Swahili or Luo—in private spaces in each health facility—and lasted about an hour. The structured responses were directly entered into the REDCap application during the interview. Interviews were also audio-recorded and transcribed (with simultaneous translation where necessary).

### Measures for quantitative analysis

#### Dependent (outcome) variables

Our dependent variables are from several survey questions on birth companionship during: (1) labor, (2) delivery, and (3) post-delivery. These include questions on whether they were accompanied by a companion to the facility, their relationship to the companion, whether they were allowed continuous support during labor and during delivery, whether they desired companionship during labor, delivery, and after delivery, and their preferred type of companion. The variables are described in Table 1 with exact wording of questions in Additional file 1.

**Table 1:** Description of Birth Companionship variables

Variable	Description
Availability of a companion	Whether or not someone accompanied the woman from home to the health facility. In addition, we asked if this person stayed with them during labor, delivery, and/or after delivery.
Type of companion	Relationship to the person who accompanied them to the facility. Women were allowed to mention more than one person, and thus, the preferred companion analysis is based on whether or not a particular type of person was mentioned.
Allowed continuous support during labor	Whether or not they were allowed to have someone they wanted to stay with them during labor. A response of “yes, most of the time,” or “yes, all the time” is coded as being allowed continuous labor support, and “no, never” or “yes, a few times” is coded as not being allowed continuous labor support.
Allowed continuous support during delivery	Whether they were allowed to have someone they wanted to stay with them during delivery. Recoded similarly to “allowed continuous support during labor”
Desire for companionship during labor, delivery, and after delivery	Whether or not they wanted the person who accompanied them to stay with them during labor, delivery, and/or after delivery; and if they will want a companion at each stage if they were to have another baby in the future
Preferred type of companion	The type of person they would want as a companion during labor, delivery, and/or after delivery if they were to have another baby. They could mention more than one person, so responses refer to whether or not they mentioned a particular type of person

#### Independent variables

These include various socio-demographic, facility, and provider related factors that might be associated with the dependent variables. These were selected based on existing literature and theoretical reasoning. They include age, marital status, parity, education, literacy, employment, household wealth, tribe, religion, prior facility delivery, antenatal attendance, pregnancy complications, labor and delivery complications, type of delivery facility, type and sex of delivery providers, and women’s perceptions of crowding in the facility. In multivariate analysis, we controlled for potential reporting bias by place of interview and post-partum length.

### Data analysis

#### Quantitative analysis

First, we conducted descriptive analysis to examine the characteristics of the sample and to assess the prevalence of birth companionship and CSLD, as well the proportion of women who desired companionship and their preferred companions. Next, we used cross tabulations and bivariate logistic regressions to examine the bivariate associations between the dependent and independent variables. We also examined whether the type of companion was associated with being allowed CSLD and women’s desire for companionship. Finally, we used multivariate logistic regression to examine the factors associated with being allowed CSLD, net of other factors.

#### Qualitative analysis

Using the Braun & Clarke approach (2006), we analyzed the qualitative data thematically to identify patterns within data [37]. We coded data inductively, considering both the semantic (surface) and latent (underlying) meaning of the text, focusing on salience rather than frequency. We iteratively read and re-read the transcripts and coded line-by-line across the entire data set. Two coders (the first and second authors) double coded half of the transcripts and compared codes to check consistency. We then analyzed initial codes to generate categories and identify themes [37]. We again reviewed transcripts until no new themes emerged. See Additional file 2 for how open codes were used to generate categories and themes. This was however an iterative process with constant reviews and revisions. Throughout the process, we wrote analytic and reflexive memos to capture emerging ideas and examine our assumptions, preconceptions, and reactions to the data. For example, we started the study with the preconception that all women will be unhappy about being denied companions. We were thus surprised by some women’s reactions to not being allowed companions and the many reasons they gave for not desiring companions. We therefore wrote a memo on this early on to ensure we adequately captured women’s perceptions. We managed data using Atlas.ti. (COREQ checklist in Additional file 3).

## Results

### Results from interviews with women

#### Univariate distributions

Table 2 shows demographic characteristics of both survey and focus group respondents. Focus group participants were older and more likely to be married, had higher parity, and lower education compared to survey participants. About two-thirds of respondents in both samples belonged to the dominant tribe of the County—the Luo tribe.

**Table 2:** Demographic characteristics of Study Participants, PQCC Study 2016

	Survey respondents		FGD respondents	
	No.	%	No.	%
Total N	877	100.0	58	100
Age				
15 to 19 years	162	18.5	4	6.9
20 to 29 years	511	58.3	38	65.5
30 to 48 years	204	23.3	16	27.6
Marital status				
Single	140	16.0	5	8.6
Partnered/Cohabiting	3	0.3	1	1.7
Married	687	78.3	51	87.9
Widowed	35	4.0	1	1.7
Divorced/Separated	12	1.4		
Number of births				
1.0	290	33.1	6	10.3
2.0	185	21.1	8	13.8
3.0	163	18.6	11	19
4 or more	239	27.3	33	56.9
Education				
No school/Primary	495	56.4	46	79.3
Post-primary/Vocational/Secondary	271	30.9	10	17.2
College or above	111	12.7	2	3.4
Literacy: writing				
No, cannot write	31	3.5	1	1.7
Yes, but with some difficulty	141	16.1	38	65.5
Yes, Very well	705	80.4	19	32.8
Literacy: reading				
No, cannot read	37	4.2	4	6.9
Yes, but with some difficulty	129	14.7	34	58.6
Yes, Very well	711	81.1	20	34.5
Employed				
No	658	75.0	39	67.2
Yes	219	25.0	19	32.8
Wealth Quintile				
Poorest	190	21.7		
Poorer	190	21.7		
Middle	135	15.4		
Richer	172	19.6		
Richest	190	21.7		
Delivery facility type				
Gov’t Hospital	404	46.1	27	49.1
Gov’t Health Center	362	41.3	23	41.8
Mission/Private facility	111	12.7	5	9.1
Delivery provider				
Nurse/Midwife	656	74.8		
Doctor	83	9.5		
Clinical Officer	54	6.2		
Non-skilled attendant	21	2.4		
1plus skilled providers	63	7.2		
Delivery Provider sex				
Male	329	37.5		
Female	514	58.6		
Both	34	3.9		
Labor/Delivery complications				
No	721	82.21		
Yes	156	17.79		
Past facility delivery				
No	342	39.0	11	19
Yes	535	61.0	47	81
Trimester of first ANC				
First trimester	261	29.8		
Second trimester	536	61.3		
Third Trimester	72	8.2		
No ANC	6	0.7		
Number of ANC visits				
No ANC	6	0.7		
Less than 4	281	32.2		
4 or 5	485	55.6		
6 plus	100	11.5		
Tribe				
Luo	584	66.6	38	65.5
Kuria	208	23.7	12	20.7
Other	85	9.7	8	13.8
Religion				
Catholic	242	27.6	12	20.7
Protestant/Pentecostal	191	21.8	22	37.9
Seventh Day Adventist	263	30.0	14	24.1
Other Christian	166	18.9	7	12.1
Muslim/other religion	15	1.7	3	5.2
Postpartum length				
Less than 1 week	75	8.6	1	1.7
1 week or more	802	91.4	57	98.3
Place of Interview				
Health facility	356	40.6	10	17.2
In the community/a home	521	59.4	48	82.8

Blanks were not asked of FGD respondents; PQCC is Perceived Quality of Care during Childbirth

The distribution of the birth companionship variables is shown in Table 3. Among survey respondents, 88% were accompanied by someone to the health facility. About a third of respondents were accompanied by a partner (29%) or mother-in-law (28%). Seventy-eight percent, 26, and 88% reported the person who accompanied them (subsequently referred to as the companion) stayed with them for some period during labor, delivery, and after delivery respectively. However, only 67 and 29% of the respondents were allowed continuous support during labor and delivery respectively. Besides, 18, 63, and 6% did not want their companion to stay with them during labor, delivery, and after delivery respectively. Also, 33, 69, and 10% reported that if they were to have another baby, they would not want a companion during labor, delivery, and after delivery respectively. For preferred companion in the future, 22% wanted their partner during labor and 43% wanted him after delivery, but only 6% wanted him during the delivery.

**Table 3:** Distribution of Birth Companionship variables, PQCC Study 2016

Birth Companionship Variables	No.	%	No.	%	No.	%
Accompanied from home to facility						
No	106	12.4				
Yes	751	87.6				
Accompanied by						
Partner-Husband	251	29.3				
Mother-in-law	236	27.5				
Mother	100	11.7				
Sister/Sister-in-law	164	19.1				
Friend/Neighbor	112	13.1				
Other ^a^	74	8.7				
Companion stayed during	Labor		Delivery		Post-delivery	
No	157	20.9	556	74	86	11.5
Yes	588	78.3	192	25.6	661	88
Don’t know	6	0.8	3	0.4	4	0.5
Companion allowed during	Labor		Delivery			
No, never	162	18.9	524	61.1		
Yes, a few times	103	12	72	8.4		
Yes, most of the time	230	26.8	110	12.8		
Yes, all the time	357	41.7	138	16.1		
Wanted Companion to stay during	Labor		Delivery		Post-delivery	
No	138	18.4	476	63.4	48	6.4
Yes	608	81	267	35.6	701	93.3
Don’t know	5	0.7	8	1.1	2	0.3
Will want companion in future during	Labor		Delivery		Post-delivery	
No	285	33.3	592	69.1	85	9.9
Yes	560	65.3	254	29.6	761	88.8
Don’t know	12	1.4	11	1.3	11	1.3
Preferred companion type in future during	Labor		Delivery		Post-delivery	
Partner-Husband	188	21.9	49	5.7	370	43.2
Mother in Law	234	27.3	119	13.9	362	42.2
Mother	91	10.6	42	4.9	138	16.1
Sister/Sister in law	165	19.3	63	7.4	244	28.5
Friend/Neighbor	77	9	31	3.6	159	18.6
Community Health Volunteer	4	0.5	4	0.5	8	0.9
Nurse/Midwife	35	4.1	21	2.5	49	5.7
Doctor	10	1.2	10	1.2	14	1.6
Other	23	2.7	8	0.9	26	3
Acceptability of being denied a companion						
Unacceptable in all instances	722	84.2				
Acceptable in certain instances	112	13.1				
Acceptable in all instances	23	2.7				
Total	857	100	857	100	857	100

^a^Other includes co-wives, grand-mother, community health volunteers, etc.

#### Bivariate results

The bivariate associations between the independent and dependent variables are shown in Tables 4, 5, and 6. We find that younger and primiparous women who had no past facility delivery, as well as women from the Kuria tribe, women with four or more antenatal visits, and women who delivered in higher level facilities were more likely (than their reference groups) to be accompanied to the health facility (Table 4).

**Table 4:** Bivariate distribution of selected birth companionship variables by demographic and facility characteristics, PQCC Study 2016

	Accompanied to facility		Allowed continuous support during			
			Labor		Delivery	
	No.	%	No.	%	No.	%
Age						
15 to 19 years	156	96.3**	118	72.8	58	35.8
20 to 29 years	442	86.5	352	69.2	153	29.9
30 to 48 years	172	84.3	130	63.7	56	27.5
Marital Status						
Not married	172	90.5	137	72.1	69	36.3*
Currently married	598	87	463	67.6	198	28.8
Number of births						
1.0	274	94.5***	216	74.5**	102	35.2
2.0	163	88.1	134	73.2	51	27.6
3.0	137	84	103	63.2	53	32.5
4 or more	196	82	147	61.5	61	25.5
Education						
No school/Primary	425	85.9	323	65.4*	132	26.7*
Post-primary/vocational/Secondary	246	90.8	189	70	99	36.5
College or above	99	89.2	88	79.3	36	32.4
Literacy						
No, cannot write	28	90.3	13	41.9***	7	22.6***
Yes, but with some difficulty	122	86.5	84	59.6	20	14.2
Yes, Very well	620	87.9	503	71.6	240	34
Household wealth						
Poorest/poorer	332	87.4	236	62.3**	105	27.6*
Middle	122	90.4	93	68.9	37	27.4
Richer/richest	316	87.3	271	75.1	125	34.5
Employed						
No	576	87.5	427	65.1***	198	30.1
Yes	194	88.6	173	79	69	31.5
Pregnancy complication						
No	431	87.2	349	70.8	168	34**
Yes	339	88.5	251	65.7	99	25.8
Birth Complication						
No	633	87.8	515	71.6***	242	33.6***
Yes	137	87.8	85	54.5	25	16
Past health facility delivery						
No	315	92.1**	242	70.8	117	34.2
Yes	455	85	358	67.2	150	28
ANC visits						
Less than 4	240	83.6**	200	69.7	92	32.1
4 plus	526	89.9	396	67.8	173	29.6
Tribe						
Luo	496	84.9***	426	73.1***	189	32.4
Kuria	200	96.2	117	56.5	53	25.5
Other	74	87.1	57	67.1	25	29.4
Religion						
Catholic	215	88.8	175	72.6	68	28.1
Protestant/Pentecostal	165	86.4	122	63.9	53	27.7
Seventh Day Adventist	236	89.7	182	69.5	94	35.7
Other Christian	142	85.5	112	67.5	46	27.7
Muslim/other religion	12	80	9	60	6	40
Delivery facility type						
Gov’t Hospital	369	91.3**	257	63.9**	115	28.5
Gov’t Health Center	303	83.7	273	75.4	114	31.5
Mission/Private facility	98	88.3	70	63.1	38	34.2
Delivery provider						
Nurse/Midwife	578	88.1	462	70.6**	209	31.9
Doctor/Clinical Officer	119	86.9	89	65	40	29.2
Non-skilled attendant	17	81	17	81	8	38.1
1plus skilled providers	56	88.9	32	50.8	10	15.9
Delivery Provider sex						
Male	294	89.4	206	62.6*	82	24.9*
Female	445	86.6	370	72.3	173	33.7
Both	31	91.2	24	70.6	12	35.3
Accompanied by						
Partner						
No			434	69.7	208	33.3**
Yes			166	65.9	59	23.3
Mother-in-law						
No			442	70	186	29.4
Yes			158	64.8	81	33.1
Mother						
No			525	67.9	231	29.8
Yes			75	73.5	36	35.3
Sister/Sister in law						
No			465	65.7***	223	31.4
Yes			135	80.8	44	26.3
Friend/Neighbor						
No			519	68.2	238	31.2
Yes			81	71.1	29	25.4
Facility crowded						
No or a few times			425	72.2**	172	29.1
Most or all the time			173	60.9	94	33.1
Desired labor companion						
No			68	47.9***		
Yes			459	73.9		
Don’t know			3	60		
Desired delivery companion						
No					92	18.8***
Yes					135	49.6
Don’t know					4	50
Total	770	87.8	600	68.6	267	30.4

* *p* < 0.05 ** *p* < 0.01 *** *p* < 0.001

**Table 5:** Bivariate distribution of selected birth companionship variables by demographic and facility characteristics, PQCC study 2016

	Desire for labor support				Desire for delivery support			
	Last birth		Future birth		Last birth		Future birth	
	No.	%	No.	%	No.	%	No.	%
Age								
15 to 19 years	128	82.1	108	66.7*	73	46.8*	58	35.8
20 to 29 years	352	79.6	327	64	144	32.6	137	26.9
30 to 48 years	142	82.6	139	68.1	55	32	62	30.4
Marital Status								
Not married	149	86.6	125	65.8**	81	47.1**	65	34.2*
Currently married	473	79.1	449	65.4	191	31.9	192	28
Number of births								
1.0	233	85	190	65.5	122	44.5**	108	37.2*
2.0	134	82.2	132	71.4	55	33.7	51	27.7
3.0	105	76.6	103	63.2	38	27.7	37	22.7
4 or more	150	76.5	149	62.3	57	29.1	61	25.5
Education								
No school/Primary	336	79.1	321	64.8	137	32.2	141	28.5
Post-primary/Vocational/Secondary	202	82.1	182	67.2	99	40.2	78	28.8
College or above	84	84.8	71	64	36	36.4	38	34.2
Literacy								
No, cannot write	22	78.6	22	71	9	32.1*	6	19.4
Yes, but with some difficulty	94	77	98	69.5	28	23	38	27
Yes, Very well	506	81.6	454	64.4	235	37.9	213	30.3
Household wealth								
Poorest/poorer	260	78.3	251	66.1	103	31**	104	27.4
Middle	98	80.3	86	63.7	39	32	33	24.4
Richer/richest	264	83.5	237	65.5	130	41.1	120	33.1
Employed								
No	452	78.5*	402	61.1***	200	34.7	184	28
Yes	170	87.6	172	78.5	72	37.1	73	33.3
Pregnancy complication								
No	346	80.3	309	62.6*	149	34.6	144	29.1
Yes	276	81.4	265	69.2	123	36.3	113	29.6
Birth Complication								
No	517	81.7	468	64.9	224	35.4	210	29.2
Yes	105	76.6	106	67.9	48	35	47	30.1
Past health facility delivery								
No	265	84.1	225	65.8	140	44.4***	122	35.7**
Yes	357	78.5	349	65.2	132	29	135	25.3
ANC visits								
Less than 4	196	81.7	190	66.2	86	35.8	91	31.8
4 plus	423	80.4	380	65	183	34.8	163	27.9
Tribe								
Luo	407	82.1	370	63.4	167	33.7	143	24.5***
Kuria	160	80	146	70.2	71	35.5	79	38
Other	55	74.3	58	68.2	34	45.9	35	41.2
Religion								
Catholic	180	83.7	155	64	73	34	72	29.8**
Protestant/Pentecostal	129	78.2	125	65.4	72	43.6	77	40.5
Seventh Day Adventist	196	83.1	184	70	79	33.5	73	27.8
Other Christian	108	76.1	102	61.4	44	31	33	19.9
Muslim/other religion	9	75	8	53.3	4	33.3	2	13.3
Delivery facility type								
Gov’t Hospital	294	79.7	256	63.4	133	36	130	32.3
Gov’t Health Center	251	82.8	248	68.5	100	33	89	24.6
Mission/Private facility	77	78.6	70	63.1	39	39.8	38	34.2
Delivery provider								
Nurse/Midwife	472	81.7	438	66.8	211	36.5	203	31
Doctor/Clinical Officer	94	79	84	61.3	35	29.4	29	21.2
Non-skilled attendant	13	76.5	14	66.7	6	35.3	6	28.6
1plus skilled providers	43	76.8	38	60.3	20	35.7	19	30.2
Delivery Provider sex								
Male	235	79.9	215	65.3	108	36.7	104	31.6
Female	363	81.6	337	65.6	152	34.2	141	27.5
Both	24	77.4	22	64.7	12	38.7	12	35.3
Accompanied by								
Partner								
No	443	85.7***	412	66	209	40.4***	195	31.3*
Yes	179	70.8	162	64	63	24.9	62	24.5
Mother-in-law								
No	419	79.8	401	63.4	180	34.3	168	26.6*
Yes	203	82.9	173	70.6	92	37.6	89	36.5
Mother								
No	534	79.9	504	65*	224	33.5*	221	28.6
Yes	88	86.3	70	68.6	48	47.1	36	35.3
Sister/Sister in law								
No	476	78.9*	456	64.2	212	35.2	206	29.1
Yes	146	87.4	118	70.7	60	35.9	51	30.5
Friend/Neighbor								
No	532	81.1	504	66.1	222	33.8*	222	29.1
Yes	90	78.9	70	61.4	50	43.9	35	30.7
Facility crowded								
No or a few times	428	83.4*	400	67.7	185	36.1	174	29.4
Most or all the time	192	75.3	172	60.6	86	33.7	82	28.9
Allowed continuous labor support								
No or a few times	162	68.1***	146	53.1***				
Most or all the time	459	86.6	428	71.3				
Allowed continuous delivery support								
No or a few times					137	25.4***	139	22.8***
Most or all the time					135	58.4	118	44.2
Total	622	80.8	574	65.5	272	35.3	257	29.3

* *p* < 0.05 ** *p* < 0.01 *** *p* < 0.001

**Table 6:** Bivariate distribution of selected birth companionship variables by demographic and facility characteristics, PQCC Study 2016

	Accompanied by Partner			Accompanied by mother-in-law		
	No.	%	*p*-value	No.	%	*p*-value
Age						
15 to 19 years	26	16	***	43	26.5	
20 to 29 years	154	30.1		150	29.4	
30 to 48 years	73	35.8		52	25.5	
Marital Status						
Not married	12	6.3	***	21	11.1	***
Currently married	241	35.1		224	32.6	
Number of births						
1.0	57	19.7	***	67	23.1	*
2.0	70	37.8		62	33.5	
3.0	50	30.7		53	32.5	
4 or more	76	31.8		63	26.4	
Education						
No school/Primary	117	23.6	***	150	30.3	*
Post-primary/vocational/Secondary	86	31.7		76	28	
College or above	50	45		19	17.1	
Literacy						
No, cannot write	11	35.5		11	35.5	
Yes, but with some difficulty	39	27.7		40	28.4	
Yes, Very well	203	28.8		194	27.5	
Household wealth						
Poorest/poorer	93	24.5	*	135	35.5	***
Middle	40	29.6		33	24.4	
Richer/richest	120	33.1		77	21.3	
Employed						
No	176	26.7	*	177	26.9	
Yes	77	35.2		68	31.1	
Has Health Insurance						
No	175	24.2	***	207	28.7	
Yes	78	50.3		38	24.5	
Pregnancy complication						
No	149	30.2		131	26.5	
Yes	104	27.2		114	29.8	
Birth Complication						
No	204	28.3		202	28	
Yes	49	31.4		43	27.6	
Past health facility delivery						
No	68	19.9	***	83	24.3	
Yes	185	34.6		162	30.3	
ANC visits						
Less than 4	58	20.2	***	85	29.6	
4 plus	192	32.8		158	27	
Tribe						
Luo	176	30.1		114	19.5	***
Kuria	53	25.5		114	54.8	
Other	24	28.2		17	20	
Religion						
Catholic	72	29.8		68	28.1	
Protestant/Pentecostal	46	24.1		59	30.9	
Seventh Day Adventist	84	31.9		75	28.5	
Other Christian	48	28.9		39	23.5	
Muslim/other religion	3	20		4	26.7	
Delivery facility type						
Gov’t Hospital	120	29.7		122	30.2	
Gov’t HC/Disp	94	26		96	26.5	
Mission/Private facility	39	35.1		27	24.3	
Delivery provider						
Nurse/Midwife	190	29		188	28.7	
Doctor/Clinical Officer	40	29.2		35	25.5	
Non-skilled attendant	6	28.6		5	23.8	
1plus skilled providers	17	27		17	27	
Delivery Provider sex						
Male	100	30.4		97	29.5	
Female	142	27.6		138	26.8	
Both	11	32.4		10	29.4	
Total	253	28.8		245	27.9	

* *p* < 0.05 ** *p* < 0.01 *** *p* < 0.001

##### Continuous labor and delivery support

Younger, wealthier, employed, college educated, and literate women were more likely to be allowed continuous labor support. On the other hand, women who were of the Kuria tribe, had birth complications, reported the facility was over crowded most of the time, delivered in hospitals, and those who did not want their companion to stay with them, were less likely to be allowed continuous labor support. Many of the factors associated with continuous delivery support in bivariate analysis are similar to those for continuous labor support (Table 4).

##### Desire for labor and delivery support

Employed women and women accompanied by a sister or sister-in-law were more likely to desire companionship during labor. In addition, younger, unmarried, primiparous, literate, and wealthier women, as well as those accompanied by a mother or a non-relative were more likely to desire companionship at the time of delivery. Women who were accompanied by a partner were less likely to desire companionship during labor and delivery. Also, women who perceived the health facility to be over crowded most of the time were less likely to desire labor companionship. Women who were allowed CSLD at their last birth were more likely to desire it in the future (Table 5)*.*

##### Preferred companion

Wealthier and more educated women were more likely to be accompanied by their partners and to desire labor companionship from their partners in the future. They were also less likely to be accompanied by their mothers-in-law and to desire companionship from their mothers-in-law in the future. Kuria women were more likely to be accompanied by their mothers-in-law and to desire companionship from their mothers-in-law during labor in the future (Table 6).

#### Multivariate results

Table 7 shows multivariate logistic regressions of continuous labor and delivery support on various predictors. The multivariate results for desire for companionship and preference for male partners are shown in Additional file 4.

**Table 7:** Multivariate logistic regression of continuous support during labor and delivery on potential predictors, PQCC study 2016

	Allowed Continuous support during					
	Labor			Delivery		
	OR	95% CI		OR	95% CI	
Age						
15 to 19 years	1	[1	1]	1	[1	1]
20 to 29 years	0.79	[0.46	1.35]	0.86	[0.50	1.47]
30 to 48 years	0.74	[0.36	1.54]	0.92	[0.43	1.95]
Currently married	1.35	[0.79	2.29]	0.98	[0.57	1.69]
Number of births						
1	1	[1	1]	1	[1	1]
2	0.61	[0.26	1.43]	1.24	[0.51	3.04]
3	0.37*	[0.15	0.95]	2.06	[0.77	5.50]
4 or more	0.39	[0.15	1.01]	1.45	[0.53	3.93]
Education						
No school/Primary	1	[1	1]	1	[1	1]
Post-primary/vocational/Secondary	0.73	[0.47	1.14]	1.27	[0.83	1.96]
College or above	0.75	[0.36	1.56]	1.21	[0.61	2.39]
Literacy						
No, cannot write	1	[1	1]	1	[1	1]
Yes, but with some difficulty	1.9	[0.72	5.05]	0.73	[0.22	2.42]
Yes, very well	2.89*	[1.13	7.37]	1.92	[0.63	5.90]
Household wealth						
Poorest/poorer	1	[1	1]	1	[1	1]
Middle	1.73*	[1.04	2.90]	0.81	[0.47	1.40]
Richer/richest	1.91**	[1.20	3.03]	1.16	[0.74	1.84]
Employed	1.97**	[1.24	3.12]	0.91	[0.58	1.42]
Past health facility delivery	2.19*	[1.05	4.57]	0.71	[0.32	1.58]
4plus ANC visits	0.68	[0.46	1.01]	0.82	[0.56	1.20]
Birth Complication	0.67	[0.43	1.04]	0.34***	[0.19	0.60]
Tribe						
Luo	1	[1	1]	1	[1	1]
Kuria	0.57*	[0.37	0.90]	0.79	[0.48	1.29]
Other	0.79	[0.44	1.43]	0.79	[0.43	1.45]
Religion						
Catholic	1	[1	1]	1	[1	1]
Protestant/Pentecostal	0.68	[0.41	1.12]	1.06	[0.62	1.79]
Seventh Day Adventist	0.7	[0.44	1.12]	1.55	[0.99	2.44]
Other Christian	0.79	[0.46	1.35]	0.93	[0.54	1.59]
Muslim/other religion	0.86	[0.21	3.44]	1.8	[0.43	7.52]
Delivery facility type						
Gov’t Hospital	1	[1	1]	1	[1	1]
Gov’t Health Center	1.98***	[1.32	2.97]	1.46	[0.98	2.17]
Mission/Private facility	0.86	[0.49	1.51]	1.67	[0.95	2.95]
Delivery provider						
Nurse/Midwife	1	[1	1]	1	[1	1]
Doctor/Clinical Officer	0.8	[0.46	1.39]	1.13	[0.63	2.00]
Non-skilled attendant	1	[0.28	3.60]	0.88	[0.28	2.79]
1plus skilled providers	0.27**	[0.12	0.63]	0.19**	[0.060	0.59]
Delivery Provider sex						
Male	1	[1	1]	1	[1	1]
Female	1.24	[0.82	1.89]	1.42	[0.92	2.19]
Both	4.68**	[1.54	14.2]	5.40**	[1.67	17.5]
Accompanied by						
Partner	0.81	[0.53	1.24]	0.61*	[0.39	0.97]
Mother-in-law	0.92	[0.59	1.44]	1.19	[0.74	1.91]
Mother	1.3	[0.70	2.42]	1.01	[0.54	1.90]
Sister/Sister in law	1.85*	[1.13	3.01]	0.73	[0.45	1.18]
Friend- Neighbor	0.87	[0.51	1.46]	0.61	[0.35	1.06]
Facility crowded	0.65*	[0.45	0.93]	1.69**	[1.15	2.50]
Desired labor companion	1.42*	[1.01	2.00]			
Desired delivery companion				1.97***	[1.48	2.62]
Interviews in community (ref = HF)	1.02	[0.70	1.49]	0.79	[0.54	1.14]
Postpartum length= > 1 week (ref < 1	0.99	[0.53	1.83]	1.24	[0.64	2.38]
Constant	0.87	[0.22	3.42]	0.15*	[0.033	0.64]
N	763			764		

* *p* < 0.05 ** *p* < 0.01 *** *p* < 0.001

##### Continuous labor support

Wealthier, employed, and literate women had close to two times higher odds of being allowed continuous labor support than the poorest, unemployed, and illiterate women respectively; and Kuria women had about 40% lower odds of being allowed continuous labor support than Luo women. Also, those who delivered in a health center and were accompanied by a sister or sister-in-law had close to two times higher odds of being allowed continuous labor support than those who delivered in hospitals and those not accompanied by a sister or sister-in-law respectively. Women who desired a labor companion had about 40% higher odds of being allowed continuous labor support than those who did not desire one. Additionally, women who reported the facility was over crowded most or all the time had about 35% lower odds of being allowed continuous labor support than those who said it was not crowded or crowded only a few times.

##### Continuous delivery support

Women had lower odds of being allowed continuous delivery support if they had a birth complication and if there were two or more skilled providers present at the delivery than if they had no complication and were assisted by only a nurse or midwife respectively. In spite of the fact that there was no significant association between being allowed continuous delivery support and perception of overcrowding in the bivariate analysis, this association became significant in the multivariate model, but in an unexpected direction—women who reported that the health facility was overcrowded most or all the time had about 69% higher odds of being allowed continuous delivery support than those who perceived that the facility was not overcrowded. Also, women accompanied by a partner had about 40% lower odds of being allowed continuous delivery support than those not accompanied by a partner; and those who desired a companion at delivery had close to two times higher odds of being allowed continuous delivery support.

### Women’s perspectives on labor and delivery support: Qualitative data from FGDs with women

The overarching themes from the analysis of the data from FGD with women are on their feelings about being allowed or denied a companion during labor and delivery, reasons why they do or do not want a companion, and their preferences for different types of companions.

#### Feelings about being allowed or denied a companion during labor and delivery

Most women were happy when their companions were allowed to stay with them during labor, and unhappy when they were not allowed to stay with them.

*“They allowed the relatives to be with us ….I felt good because there are times you can be going to bath, and so they will remain and take care of the baby for you.” (KP5)*

*“No they didn’t [allow relative to stay] and I did not like it because they are busy with their own things and it is this relative of yours who can help you with your problem…” (WP1)*

Companions were however only allowed at certain times, like during labor but not at the time of delivery, or only after the baby was born; or during certain times of the day like at night, but not in the morning. Many felt it was inappropriate to not be allowed a companion during labor and after delivery.

*“I wanted her to be with me so that I can explain to her how am feeling so that in case I need help then I send her to go and call the doctor or nurse to come and help me quickly and they kept on telling them that “get out care takers your time is not now, wait until they give birth is when you can come in”, and yet after giving birth am not as serious as when I still have my labor pains when am not supposed to be alone and so I felt bad.” (WP2)*

However, many did not mind not being allowed a companion at the time of delivery for various reasons described in later sections.

*“I felt it was good if they were allowed to be with me after having my baby. But before, it was good that they were not allowed to be with me.” (CP10)*

It appeared some providers did not take time to explain to women about why they were not allowed a birth companion, and were sometimes abusive when disallowing the companion.

*“They refused and even chased the person who accompanied me and told her ‘leave her alone …. you did not give her the pregnancy she will deliver it herself’…. that was not good.” (MP2)*

Women therefore inferred various reasons why their companions were not allowed to stay with them. Some felt that it was a facility requirement that applied to everyone. However, others felt that it was the providers who did not want them to have someone close to them.

*“They refused so I just stayed alone in the hospital until I stayed two days in the hospital… It is required that I just stay alone…. I felt bad” (MP4)*

*“They do not always allow somebody to be with you, it is the doctor and you alone. Whoever has brought you is to wait outside” (OP5)*

*“They do not want someone to be close to you, even after giving birth, they tell them that time for visiting patients is over and they send them away.” (CP10)*

Some women also thought companions were less likely to be allowed during delivery if they were male compared to if they were female companions.

*“I was allowed to be with a woman relative but not a man… The woman relative was allowed for when there is a problem she can assist the nurse, but a male relative they do not allow them to stay.” (P10)*

#### Reasons for desiring a companion

##### Instrumental support

The most common reason for desiring a labor companion was to have someone readily available to attend to their needs. This was important because they often felt helpless in labor, and providers were not with them all the time. Also, because providers were usually busy with other things, the presence of their companion ensured that there was someone readily available to help them if they needed help for things such as going to the bathroom room and to call the provider if they developed a problem or went into the second stage of labor.

*“They did not allow them just as the other person has said, you want to be with your relative and they are sent out. You know sometimes they the doctors are out and have gone wherever places. For you your labor is bad and you are bleeding, so the energy that you will have to walk and go to call the doctor you do not have and so it is your relative whom you can send to go and get you the doctor and so that is why I was not happy with this.” (WP6)*

*“I feel it is good to have someone with you because sister [the nurse] can show you the bed to go and sleep, then she goes to sit somewhere else. So if you are about to give birth then you can send her to go and call for you the nurse so that she can come and help you.” (CP4)*

Companions were also needed to help run errands: like going to buy drugs and supplies that might not be available on the ward or arranging additional help if they were referred.

*“ I feel that they should be next to you because, the doctor can tell you that there is some drug that you should be treated with, and so he can be sent to go to the chemist to go and buy.” (CP1)*

*“They should be around because sometimes you can develop complications and you are being referred and so if you are alone then you may not be able to get help.” (OP6)*

In addition, companions were needed after delivery to help care for the baby, including holding the baby when they needed to go to the bathroom.

*“They should be allowed. Because they can help you with carrying the baby.” (CP10)*

*“I felt good because there are times you can be going to bath, and so they will remain and take care of the baby for you.” (KP5)*

##### Informational support and advocacy

For others, the companion helped meet their informational needs by telling them what to do, helping them make decisions, and advocating for their care—as they felt they were not in a state to make decisions on their own during labor. Others wanted the companion around in order to have someone who could remind them later of what happened during labor.

*“They should allow your relatives to be with you at that particular time in case that anything happens then they can share with the nurses to know what next is to be done to you, because at that time, you are in so much pain that you cannot make any wise decision.” (KP3)*

*“For me I like it because at that time I do not know anything that am doing so when she is there then she will be able to tell me that at that time you did this and that until the doctor either did this to you is when you were able to put to birth.” (OP3)*

##### Emotional support

Emotional support was barely mentioned as a reason for desiring a companion. Only one woman’s response to why she was unhappy about being denied a companion related to a desire for emotional support during labor.

*“That was not good because when you have someone near, she will try to make your heart relax, yes, but when they refuse you are just alone you will surely have some problems.” (MP2)*

#### Reasons for not desiring for a companion

Some women did not desire companionship during labor, and in particular at the time of delivery, and so did not mind that their companions were not allowed to stay. Reasons for not desiring companionship included the following.

##### They cannot help you

A common reason women gave for not desiring a companion was that “they can’t help you.” Some mentioned that the reason why they come to deliver in the facility was because their relatives could not help them at home, and so there was no need for them to be around once they had brought them to the facility.

*“I do not want because when you left home…they could not help you and so that is why you came to get help from the hospital.” (CP4)*

Others felt it was only the provider who could help them, and that they felt safe with the providers. Thus, there was no need for their companion to be around.

*“I feel that it is the right thing [that companion was asked to wait outside] because whoever has brought you is not the doctor, and he has brought you in safe hands of the doctor and so he should just stay out and wait …. because there is nothing he will do apart from just looking at you and so it is only when the doctor is not able then he will refer you.” (OP4)*

In particular at the time of delivery, many felt it was the provider who knew everything and so should be the only one with them.

*“It is the doctor who knows everything and so he is the one who should be with you alone in the delivery room but the other person I would not like him to be next to me when I am giving birth.” (OP3)*

Some also felt lay companions, in addition to not being able to help them, might laugh at them if they saw their condition at the time of delivery.

*“ I should be with the doctor alone because she is the one with all the know-how but this relative of mine, all he will do is just look and laugh at you.” (WP2)*

Others felt at the time of delivery it was only “between you and your God” and so there was no need for a relative to be there to see their nakedness

*“We did not say that it is bad to have someone with you…let them just come. But when time comes that you are in a state of between you and your God, then let them stay outside and wait for you….At that time, he should not see my nakedness.” (OP3)*

##### Embarrassment

Some women felt embarrassed to have someone other than a provider see them during labor, and particularly during delivery. They also felt that providers had seen a lot to not be surprised at their condition or nakedness, but did not feel the same way about lay companions.

*“No, I feel embarrassed and [will] rather be with the doctor alone in that hospital.” (CP5)*

*“I refused because when you are giving birth, maybe you do not have clothes and you are with your mother-in-law so you feel embarrassed.” (CP8)*

*“It is better if you are with the nurse alone because for her she has seen a lot but the other person can exclaim that ‘ei and this person.” (CP10)*

Certain roles companions were asked to play made women particularly vulnerable, as well as decrease their desire for companionship. For example, one woman reported she felt very embarrassed because the provider asked the relative to hold the flashlight for him to conduct the delivery (because there was no electricity) and she felt completely exposed to this relative.

*“I did not like it because whoever escorted me is the one that the doctor made to carry for him the sport light and was pointing it at me and I did not like it at all….but it is only that I was in a problem and I was praying to God to help me, but my inner heart did not like the fact that this person was seeing my nakedness.” (OP3)*

##### Fear that support persons may go outside the facility and discuss private matters

Some feared that the support person may discuss what they saw about them with others outside the facility. They were confident that providers could not discuss what they saw about them outside the facility, but did not feel the same about lay companions.

*“At that time they should not allow someone to be in, the doctor is the only person who should see my nakedness…people are so mouthy. Some people when they leave the hospital they go and say so many strange things about you, how they saw you, but the doctor is not allowed to go out and talk about what has happened in the facility.” (KP4)*

*“For me I don’t want because they can look at you then go and tell other women that ei.. so and so is like this.” (CP2)*

*“For me I liked it and felt good [that the person was not allowed inside] because whoever is taking care of you can go out and talk about your secret but the doctor cannot.” (CP5)*

##### Fear of abuse

Some did not want the companion to stay with them because they feared that they might unknowingly abuse the support person when they are in pain.

*“After reaching with her at the door, then he should stay aside because you are very angry at that time and so you can find yourself insulting her.” (CP10)*

Others feared that the support person may abuse them—like shouting at them at them during labor pains. The fear of being abused was mentioned in relation to the type of support person. Some women felt that their mothers-in-law were more likely to be abusive, hence they preferred to have their mother as a support person.

*“it’s good to be with a parent when giving birth, like me I was with my mother when I was giving birth and I was attended to well but when you are with your mother in law, when taking care of you, she will shout at you that stop disturbing me but when you are with your mother then she will take good care of you, it’s good to be with your mother than any other person.” (CP9)*

### Reasons for not desiring birth companionship from their partner

Women spontaneously talked about preferring a female relative, and not their partners, as birth companions. Reasons for this included that their partners could not help them and so did not need to be there.

*“ He will not help you with anything…so it is better for him to be away.” (KP2)*

Others did not want to see their partners during the time of delivery because they hated him at that time.

*“ While I was giving birth, I went with my husband as my mum lives in [another town]. But my husband was waiting for me far away…. I really did hate him at that time (…Laughs…) when that time came I felt hatred for him and I told him you sit that side” (CP6)*

Another reason was fear that their partners might lose sexual desire for them if they saw them at the time of delivery. It appeared that providers reinforced this belief by using it as a reason to discourage the women from having male partners present at the time of delivery.

*“When I am inside, I would want him just to stay outside there…The time the baby is coming out, is not a good sight for a man to be there…They always say that when a woman is delivering and the time the baby is coming out, your body is not looking good so when the husband sees that, after delivery, he will not have a desire (sexual desire) for you. That is what is said and most of us mothers know about this {laughter…}. They prevent the husbands from seeing this so that later he may have a desire for you. That is why the nurse will enter inside with you only unless there is something she cannot handle is when he will be allowed in (WP4). M: This is what nurses are saying or who?{All in chorus, nurses…}”*

There were also some cultural beliefs around allowing men in the delivery room. Some mentioned their tribes required a mother-in-law to accompany the women at the time of delivery, because the presence of a man can prolong the delivery. Thus, partners were not allowed even during deliveries conducted by traditional birth attendants [TBA].

*My third child I delivered at the TBA place. I was accompanied by my husband and other mother-in-laws. When we arrived there the baby was almost coming out, he was told to stay outside [and] not to go in. This was because the Luo culture says that when your husband goes inside with you where you are going to deliver, he will tie you and the baby might delay in coming out, so it is not good that we go with our husbands to where we are delivering (WP7).*

Others did not want their partners present because they wanted them to go home and take care of other children.

*“For me in most occasions when am going to deliver, I usually go with my husband, and when we go when we reach, after I have been admitted, Like this one, they asked me whoever he was to me and I told them that he is my husband and they asked me if I wanted him to stay behind and be with me. But I told them that no the children are home alone and so he must go back home as I was in safe hands and he was to go back and only call him in case there is anything that needs his attention then they could call him come back…I did not see the point of having [him] around because when giving birth, even if he is my own husband I do not want him to be with me at that time, I want only a doctor.” (KP2)*

### Providers’ perspectives: quantitative and qualitative data from in-depth-interviews with providers

We conducted in-depth-interviews with seven clinical officers, 25 nurses and midwives, and 17 non-clinical staff including cleaners and cooks. Thirty of them worked in public hospitals, 13 in health centers, and 6 in mission hospitals (Table 8). Most of the providers reported that women were usually allowed a companion during labor: 84% reported that during labor, companions were allowed all or most of the time. Fewer, however, reported that women were usually allowed a companion during delivery: only 39% reported that during delivery, companions were allowed all or most of the time. Clinical staff were also more likely to report that women were allowed continuous delivery support compared to non-clinical staff. Additionally, compared to female providers, male providers were more likely to report that women were allowed continuous labor and delivery support (Table 8).

**Table 8:** Univariate and Bivariate distribution of Provider data, PQCC study 2016

	Characteristics of Providers		Percent reporting women are allowed continuous support during			
			Labor		Delivery	
	No.	%	No.	%	No.	%
Facility type						
Govt. Hospital	30	61.2	22	73.3	10	33.3
Govt. Health Center	13	26.5	13	100	6	46.2
Mission Hospital	6	12	6	100	3	50
Position in facility						
Clinical officer	7	14.3	7	100	4	57.1*
Nurse/Midwife	25	51	19	76	12	48
Support staff	17	34.7	15	88.2	3	17.6
Gender						
Male	14	28.6	14	100	9	64.3*
Female	35	71.4	27	77.1	10	28.6
Age						
24 to 39 years	30	61.2	26	86.7	12	40
40 to 58 years	19	38.8	15	78.9	7	36.8
Marital status						
Single/Widowed	8	17	7	87.5	3	37.5
Married	39	83	32	82	15	38.5
Parity						
0 to 3	34	72.34	28	82.4	13	38.2
4 to 7	13	27.66	11	84.6	5	38.5
Feels Facility is crowded						
No, never	14	28.6	12	85.7	5	35.7
Yes, a few times	6	12.2	5	83.3	3	50
Yes, most of the time	23	46.9	21	91.3	9	39.1
Yes, all the time	6	12.2	3	50	2	33.3
Women allowed labor support						
No, never	1	2				
Yes, a few times	6	12.2				
Yes, most of the time	12	24.5				
Yes, all the time	29	59.2				
Women allowed delivery support						
No, never	18	36.7				
Yes, a few times	12	24.5				
Yes, most of the time	9	18.4				
Yes, all the time	10	20.4				
Total	49	100	41	83.7	19	38.8

* *p* < 0.05 ** *p* < 0.01 *** *p* < 0.001

#### Role birth companions play in the facility

The reasons given by providers for allowing birth companions were generally consistent with those given by women. Providers however also implied that the companions were useful not just to the mother but to them the providers. Providers reported companions sometimes helped them with various chores, such as going to purchase supplies and drugs, calling on them to attend to the women, helping women to and from the delivery bed, holding items like a flashlight during delivery, helping them pick up delivery supplies, and cleaning up. The role of the companion was particularly useful in the face of staff shortages, where only one provider may be on duty.

*“You know after that like now we have staff shortage, so maybe after examining a mother maybe I am alone in the facility, I may run to come and see clients here but when they are almost ready, she will come and call me. Even after delivery helping them to do one, two, three, like cleaning the clothes something like that.”*

*“Most of the time we need one caretaker to be around. In case of any eventuality they can be asked to go may be to organize for example in a case of complicated delivery and you want things like gloves somebody at least you can send to get for you the things you want, so most of the times we allow one caretaker to be around the mother who is giving birth.”*

Some providers insisted that they almost always allow birth companions, because they can’t do without them when they are alone assisting with the delivery.

*“Rarely do we not allow birth companions, rarely, not unless the mother came alone. There are some who come without Birth companions though during ANC they are encouraged to have with Birth partners, but when they come without they are equally just served as others. But when they come with a birth partner, especially X facility, if they do not assist us I don’t know how we would do it, because they do assist us even wheeling the patient to the bedside post delivery they do a lot to us especially to me when I am on duty, they do a lot.”*

*“We have no problem, if the mother has allowed someone who escorted her to labor to get in then we have no problem with the person as there was a time when a mother was delivering and it is the husband who was holding for us the touch.”*

Some support staff mentioned that some clinical providers only allowed birth companions who could be helpful to them.

*“The delivery room is small so the nurse will only need the person who will assist her get things like cotton wool, bucket for the placenta and water.”*

*“The relative may only come in when it is at night and the lights are off so the care taker gets in to hold for the nurse the torch only.”*

In addition, although companions were usually not allowed at the time of delivery, they were sometimes called on to help with non-compliant women. This was mostly noted by the non-clinical staff.

*“This time is more private, he does not allow anyone to be with the mother unless he is alone and the mother is not cooperative…[that] is when he will call a relative to come and talk to the mother to push the baby.”*

*“No, it is only the nurses who are in there. The person is to wait outside. Only if the woman is difficult to deal with is when they call the caretaker to come in and help them.”*

#### Reasons why companions may not be allowed in the ward

The reasons why birth companions were sometimes not allowed in the ward are related to privacy and ward set up, distrust of companions, lack of confidence in companions in the event of complications, and respecting the woman’s wishes.

##### Privacy and ward set up

Most providers reported that companions were sometimes not allowed in the ward because of limited space and overcrowding, hence privacy concerns for other women in the labor and delivery wards. Some also mentioned that the set-up of the labor wards is not conducive for companions, including limited seating, which may cause companions to use beds meant for the laboring woman.

*If they are two in labor then now you see the privacy will [be a problem] …but if that woman is all alone there, then we will just allow, so it is more of the setup which is preventing us but we know…that if they want even their spouses to come to the delivery room they should be allowed but because of the setup and the environment now it is not really conducive.*

Privacy was the main reason given for not allowing companions at the time of delivery.

*“Yes, we allow them but it is not a normal practice here and at second stage we do not allow them. Here you find maybe they came with them, they stay with them but when we are going to second stage we tell them to wait until the time they have delivered. But that one is done because of space as I was saying. Maybe…the relative of this mother A is there and another mother B is delivering so when I bring these relatives here they will also be watching this mother B, so we say let me counsel them when we need them we can always call them after delivery”*

##### Distrust of companions

Another set of reasons for sometimes not allowing companions were related to distrust of the companions. Providers had several fears of inappropriate things companions did (or which they thought companions might do), which made them reluctant to allow companions. These included the following:

*Fear companions will practice things usually done in home deliveries like giving the laboring woman herbs:* Several providers mentioned they sometimes refuse to let the companions into the ward because some of them bring traditional herbs that are thought to hasten labor to the health facility to give to the laboring woman. They also mentioned that some companions attempt to apply fundal pressure and touch women with unclean hands.

*“like here they like coming with the herbs, since the nurse is one, you leave the mother with the care taker and by the time you are back, you find that they have place for the mother the herb, so it depends on who has brought the client.”*

*“Sometimes you find the mother in second stage, a mother comes in, there are certain things they usually practice home deliveries, so you can find that they start practicing that so we don’t allow that in health care so this is why. Mothers at home give fundal [pressure] and they can touch anywhere with bare hands, yeah so in a normal delivery procedure they remain outside the delivery room.”*

They were particularly distrustful of older birth companions, particularly mothers-in-law, whom they said were more likely to give women concoctions to try to hasten labor.

*“Mostly those old mothers, the grannies, the mother in laws who are old. They believe that once a mother comes to the labor ward to deliver, maybe to get a baby as fast as possible, so once they are around, they can discuss and give the mother some medicine to fasten delivery so [because of] that most of us who are midwives are very keen and we don’t want those mothers to come so many of them, they can give herbal medicine or they boil tea concentrated without sugar as they believe that this one will hasten the delivery. That act most of us are not ready to entertain.”*

*Fear companions will force mothers to do things against their will:* Some providers did not trust companions because they feared they may force women to do things against their will such as forcing the women to take the herbs.

*“some mothers come with the herbs and force the laboring woman to drink or chew and so that is why we don’t usually allow them to come in where the woman is”*

*Fear companions may misinform mothers:* There was also the suspicion that companions may misinform the laboring women when they are with them in the labor ward, such as asking them to push before they are fully dilated.

*“Mmm sometimes you get, okay personally, the mothers who come with a companion, give misinformation to the mothers, they force the mothers to push before they are fully dilated, and that is why many a times I do not allow them because they give a lot of misinformation to the mothers while they are in labor. [It causes] those things like cervical tear which are not supposed to be there.”*

*Fear that companions may misinterpret what they see in the facility and discuss it outside:* Providers also sometimes prevented companions from staying in the ward because of fears that when they observe certain procedures and something goes wrong (like the baby dies), the companions will go out and spread rumors that the baby died because of the procedure the provider performed.

*“When you have invasive procedure… the companion is not supposed to be around to see, for they can feel that you did that thing wrongly and yet we were helping the mother or the baby. I: Like what procedures? R1: Like vacuum extraction you cannot allow the companion to be there. There is fear they can take the information at home that they did this that killed our baby by using the instruments.”*

A support staff mentioned that clinical providers also sometimes disallowed companions because of fears that companions will go out and gossip about the woman’s condition to others, including adding their own interpretations to what they saw.

*Some women after seeing the woman delivering, they go and talk about how the woman’s private part is. Some women also give birth to babies who are white with a lot of whitish things in them, so they go and start gossiping how the woman was having sex until the last minute is why the baby is born dirty. So that is why the nurses want to do everything and clean the baby. [That] is when she is taken to the other room for the relatives and friends to see the baby.*

*Fear of companions being disrespectful to providers and not taking instructions:* Another reason some providers gave for sometimes disallowing companions was that some of them were disrespectful: they talk rudely to them, do not take instructions, want to order the providers around, and are naughty and noisy.

*“…some care takers are so stubborn they talk to the nurses as if they know everything that the nurse should be doing.”*

*“Sometimes, some people want to be the nurse and so they give the nurse orders in there. So such people are told to wait in the labor room.”*

*“…also it depends on the understanding as some of them will come and don’t want to listen to the instructions of the provider.”*

##### Disallowing companions for women with complications

Women with chronic medical conditions or pregnancy complications were sometimes not allowed companions because of the following:

*Need to keep the woman’s health information confidential:* Some providers mentioned not allowing companions to maintain the woman’s confidentiality, especially if she had a condition like HIV.

*“Like when the patient is positive (HIV) the doctor will first talk to her and find out from the patient whom to inform and not everybody because at times is a neighbor who brought her. Also find out from her who will collect the drugs for her. In such a case is only the doctor who will be with the patient until she says whom she would want to be closer to her.”*

*Need to monitor woman frequently:* Some also mentioned not allowing companions if the woman had a condition that they needed to monitor frequently.

*“Okay, sometimes if a woman has a chronic condition that you need to monitor, so we may not like a lot of relatives to be there but we have to inform the relative that we are doing this and this. So you will find at that time [only] the health worker will be so near to the patient to monitor.”*

*Inappropriate companion behavior:* Providers were also concerned that in the event of a complication, the companion’s behaviors may discourage the woman.

*“Sometimes just in case of any complication as those people, the family people, they will tend to change to discourage the patient by the way they behave. Like they are so anxious about the patient and they try to discourage.”*

*“Some relatives are so scared and so they are not allowed to get in because they will make the woman to also be scared.”*

A provider mentioned fear of companions collapsing because of what they saw, giving them additional work of caring for the companion as well as the woman.

*“There are a few cases the condition of a mother does not warrant the relatives to be near the mother because of big number like sometimes we had seen somebody who has escorted the patient after seeing the thing fainted, now you will have double work, you are struggling with the mother and somebody has just fainted. So in such scenarios in some cases we do tell some not to. Or you are seriously resuscitating a child and the person who has escorted the mother is crying, you tell her to be out as we do the resuscitation process.”*

#### Respecting the woman and companions wishes

Sometimes companions were asked to stay outside the labor or delivery ward to respect the woman’s wishes.

*“ If the mother who is in labor says, me I do not want anyone around until I deliver.* [1] *just preserve that as her right, but most of the times we just allow the relatives to be around.”*

Other times, it was the companion who did not want to stay with the woman, because some women, in their pain, were cruel to their companions.

*R: During labor pains, most of the women become very cruel or very rude. So some of the family members may be afraid to go and be with them in there. [Laughs]We tell her to choose the best person so if she does not want you then we tell you to leave.*

### Perceptions about partners as birth companions

Providers spontaneously mentioned that they were more likely to allow continuous labor and delivery support if the companion was a partner. They mentioned that partners are the first selected to support women when there is limited space, because they did not trust other companions.

*“ Mostly we allow the husband, we allow them to be with the wife but if another care taker, sometimes we don’t allow. I: Why, what is the reason? R1: Because you never know who is the caretaker”*

*“we do not know the caretaker and you know family issues we don’t interfere, only the husband we allow in the delivery room if the patient is willing, some patients are not willing; they tell the husband go away”*

They, however, acknowledged that many women did not want their partners in the delivery room because they wanted their privacy.

*“Maybe the mother will just say let him [partner] or her go outside for sometime. They do not like when they are pushing especially their husbands [to be around].”*

*“Sometimes it is the husband who has come, and the woman is the one who sends him away so we also tell them to keep off and wait outside as she is putting to birth”*

Other times, it was the partners who did not want to be in the delivery room: because they did not want to see the nakedness of women, didn’t find it appropriate to enter the room, or because of cultural reasons.

*“They are allowed, but men mostly don’t come…One said that he didn’t want to see the lady naked, he doesn’t want to see her being handled during delivery. But us we do allow them”*

*“I may say its culture, let me say if it’s a husband who has brought the wife, some of them do not want to go where the woman is laboring.”*

Some providers also discouraged partners from being birth companions, due to the perception that the presence of a partner may cause women to desire more attention.

*“when the husband is around this might affect her psychologically as when she sees the husband she might want the sympathy from him. When the husband is away she will be with the nurses who should take care of her.”*

## Discussion

This study is the first study, to our knowledge, to comprehensively examine the prevalence and nature of labor and delivery support from the perspective of both mothers and providers in Kenya, and among the first of such studies in SSA. We found that most women are accompanied to the facility for childbirth and most of them want to have a companion stay with them during labor and after delivery, but fewer want that person around at the time of delivery. Women’s reasons for desiring a companion were mostly related to having someone around to help them meet their physical needs; more so than for emotional support. Those who expressed that they did not want a companion felt that companions could not help them, were embarrassed to have the companion see their nakedness, feared that companions may gossip about what they saw, or were afraid the companion might abuse them or vice-versa. Most providers acknowledged the importance of having a birth companion but stated that it is often not possible due to privacy concerns and other reasons related to distrust and lack of confidence in companions. Some providers, however, thought of companions’ roles as assisting them with non-clinical tasks rather than providing emotional support to women.

The findings from this study support the few other studies in SSA that challenge the assumption that all women want a birth companion [13]. In addition, continuous support is often examined as a single unit, but our findings suggest that combining labor and delivery together may not align with women’s preferences. By examining labor and delivery separately we show that women have different desires for companionship at different stages of labor and delivery. Furthermore, we show that having a companion present doesn’t guarantee that the woman will be supported or feel supported. Where providers think of companions as providing support to them, women’s needs might be overlooked. Nonetheless, women who were allowed CSLD in their last pregnancy were more likely to desire it in the future, suggesting that they valued the experience.

We also extend the discussion on women’s preferences for companionship at labor and delivery. Less than a quarter of women mentioned a partner as their preferred companion during labor, and even fewer (only 6%) wanted a partner as a companion during delivery. Most women preferred a female companion, such as their mother, mother-in-law, sister, sister-in-law, or friend. A few mentioned co-wives, but notably no one mentioned a traditional birth attendant (TBA) as their preferred companion. The reasons for the low preference male partners included fear that their partners might lose sexual desire for them if they saw the baby coming out, as well as cultural beliefs about how the presence of a partner could affect the delivery. The preferred type of companion is also influenced by tribal norms, with a mother-in-law being preferred over the partner among the Kuria tribe. Our findings are consistent with that of a study in Ghana, where 24% of respondents preferred a male labor companion [13], but much lower than that from a study in Nigeria where most women (86%) preferred their husband as their labor companion [21]. A reason for this might be that the study in Nigeria involved women with higher socio-economic status than our respondents. We found that wealthier and more educated women were more likely to desire their partners as both labor and delivery companions than poorer and less educated women.

The national guideline for Obstetrics and Perinatal Care in Kenya specifically mentions partners as birth companions, and, currently, there are initiatives to promote male involvement in maternal and child health in Kenya. These may be reasons why providers spontaneously discussed a preference for partners as companions during interviews. The survey results, however, suggest that women are less likely to be allowed continuous support at delivery if the companion was a male partner. In addition, women perceive that providers are less likely to allow them to have continuous labor and delivery support if their companions are male partners. This inconsistency between providers’ and women’s report might be due to providers responding in a socially desirable manner in spite of the fact that both groups are not particularly supportive of having male partners as birth companions. Moreover, the spontaneous discussion among women about male companions may suggest that they are not entirely supportive of the emphasis on having their partners as companions. These findings suggest that we need to find better ways of changing social norms around the role of men during labor and delivery and promoting the involvement of male partners in maternal and child healthcare, while prioritizing women’s preferences.

Morhason-Bello et al., found that emotional support was the most cited reason for women’s desire for birth companionship (80%). This finding is not consistent with that of our study, and the demographics of our sample may explain the difference. The limited reference to emotional support should, however, not be misconstrued as a lack of desire for emotional support. We did not specifically ask our participants about emotional support or other types of support. The reasons women give for desiring labor companions are therefore likely the top reasons based on the realities of their situation—which in this case is a need for instrumental support to meet their practical needs at the facility. In addition, Alexander et al., in their study in Ghana, found that male companions were expected to provide emotional support, while female companions were expected to provide other types of support [13]. The limited reference to emotional support in our study may therefore be due to the low preference for male companions.

Beyond women’s desires and preferences are health system barriers to providing continuous labor and delivery support. Providers cite overcrowding and privacy issues are the main reasons for disallowing birth companions. This is consistent with our survey results, where women who perceived the facility as crowded were less likely to be allowed a labor companion. It is also consistent with findings related to barriers to promoting CLSD in other low resource settings [8]. Our findings suggest that providers sometimes have to prioritize different aspects of respectful maternity care—and they tend to choose privacy over CSLD. Overcrowding, however, did not predict continuous delivery support in the way we expected—women who felt that the facility was overcrowded were more likely to have continuous delivery support. It is unclear why this is so, but this finding highlights the importance of other factors besides crowding that may affect CSLD.

Providers gave other reasons for disallowing companions, such as mistrust and lack of confidence in companions. In contrast, women frequently expressed confidence and trust in providers, obviating the need for a companion. Providers seemed particularly distrustful of older women, and especially mothers-in-law, who are the most common companions among Kuria women. Kuria women were therefore less likely to be allowed continuous labor support, compared to Luo women, even though they were more likely to have a companion present at the facility. Providers’ preference for younger female companions is reflected in women being more likely to be allowed continuous labor support if they had a sister or sister-in-law as their companion. Furthermore, being allowed labor support is predicted by socioeconomic status (SES), with wealthier, employed, and literate women being more likely to be allowed continuous labor support than poorer, unemployed, and illiterate women. This finding is consistent with the literature on differential care in health facilities based SES [38, 39]. Additionally, women with complications were less likely to be allowed continuous labor and delivery support than those without complications. The reasons for this include, a perceived need by providers to keep women’s health information confidential, avoid interruptions during the care process, and protect women from companions who might not be helpful in supporting them to deal with complications. Again, it appears that providers feel the need to prioritize, and companionship is often not considered as a top priority.

Health system weaknesses also dictate the role of companions. The shortage of clinical and support staff and the burden placed on families to purchase medicines and supplies outside of the facility, have caused providers to redefine the role of companions. This is inferred when providers mostly mention instrumental support from companions as a reason for permitting women to have companions. The instrumental role of companions was mentioned as very useful in the face of staff shortages. This perspective may account for companions being more likely to be allowed in health centers, as there are usually fewer providers on duty in lower-level facilities. In addition, providers, just like the women, hardly mentioned emotional support as a reason for companionship. There is likely limited understanding among providers, as well as women, about what emotional support or woman-centered care should look like. These findings imply a complex interaction between women’s needs and preferences, as well as providers needs and preferences, within facilities and systems that might not be set up to provide CSLD.

Our findings have some implications for research. First, despite WHO recommendations on birth companionship, there have been very few studies on it in SSA and other low resource settings. We need rigorous studies to better understand the effectiveness of different forms of birth companionship in these settings. For example, when women have a companion present only during labor and not at delivery, does it reduce the positive effects of CSLD and by how much? Also, given most companions in this setting are relatives and not professional support persons like doulas, how can we improve the effectiveness of CSLD? Can we learn from doula programs in developed settings and develop a cadre of providers for CSLD with whom patients can feel comfortable? Can TBAs be trained as doulas and will that be acceptable to women? (noting that none of the women in our sample was accompanied by a TBA and none mentioned a TBA as their preferred companion). Given reasons for not desiring companionship at delivery, should family members be companions during labor and post-partum and another cadre like a doula be companions for delivery? Will doula programs be feasible, acceptable, and sustainable in low resource settings? What level of training for companions is sufficient to have an impact and be cost-effective for low resource settings? Given privacy concerns and larger health systems barriers, what changes can be made to promote labor and delivery support? And how can we address the concerns of both women and providers to ensure that labor and delivery support is effective and reflects their needs and concerns.

The study has some limitations. First, the data are based on self-report. Social desirability and recall bias are thus potential limitations. Second, the findings may not be generalizable to all women and providers. in Kenya, given the peculiarities of the study County. In addition, the qualitative data may not reflect the perceptions of all women in the study County. Nonetheless, the consistency of these findings with other data from the study suggests some transferability. Despite these limitations, this study makes valuable contributions to the existing literature on social support in low resource settings. It is one of the largest and most comprehensive studies on labor and delivery support in SSA.

## Conclusions

This study provides important insights that can be used to improve practice and policies on birth companionship in low-resource settings. The findings suggest that there are differences in women’s desire for companionship at different phases of labor and delivery, as well as their preference for different types of companions—and these are associated with factors such as age, SES, and tribe. Therefore, it is essential for providers to understand and respect women’s preferences and desires. For example, women may desire support during labor, delivery, and post-delivery; only during labor or post-delivery; or not at all; these preferences should be respected. It is also essential that providers identify women’s preferred companions, and that the person who takes the woman to the facility may not be her preferred companion. Antenatal care is an opportunity to help women understand the importance of emotional support and the need to have their preferred companions with them when they go to the facility to deliver. Training providers, as well as companions, could help address the distrust and lack of confidence in companions as a barrier to allowing CSLD. It will also provide more role clarity and help highlight the value of emotional support during childbirth. In general, we need better definitions of appropriate support and interventions to sensitize women, family, and providers about the role of birth companions. Finally, many health systems in low resource settings do not have the capacity to provide CSLD. Thus, we need structural interventions within health systems to ensure that labor and delivery wards are organized in ways that facilitate CSLD. These include redesigning labor and delivery wards to have adequate space to accommodate companions and partitioning them to ensure patient privacy. Addressing the shortage of health care providers and the lack of needed drugs and supplies within maternity units (which requires family members to acquire them outside the unit) will help reduce the perceived role of the companion as an aide to providers (and one needed to run errands) and increase emphasis on their emotional support role. Without system strengthening it will be difficult to provide truly woman-centered care.

## Additional files

Additional file 1: Interview guides. (DOCX 95 kb)

Additional file 2: Coding example. (DOCX 96 kb)

Additional file 3: COREQ checklist. (PDF 66 kb)

Additional file 4: Multivariate regression of desire for labor companionship and male companions on potential predictors. (DOCX 105 kb)

## Abbreviations

CSLD: Continuous support during labor and delivery
FGD: Focus group discussions
PQCC: Perceived Quality of Care during Childbirth
SSA: Sub-Saharan Africa
WHO: World Health Organization
