# Supplementary material for: Companionship during facility-based childbirth: results from a mixed-methods study with recently delivered women and providers in Kenya
Source: BMC Pregnancy Childbirth. 2018 May 10;18:150. doi: 10.1186/s12884-018-1806-1 (PMC5946503; doi:10.1186/s12884-018-1806-1)
Supplement: Supplementary file 1 — Interview guides. (DOCX 95 kb) [file 12884_2018_1806_MOESM1_ESM.docx]

**Additional file 1:**

**Birth companionship questions from PQCC study questionnaires and Interview guides**

| **Questionnaire for Surveys with women** | | | |
| --- | --- | --- | --- |
| Q No. | Field Label | Choices | Note/skips |
|  | SOCIAL SUPPORT  Now I am going to ask you some more questions to help us understand women's preferences for support persons during labor and delivery |  |  |
| 169 | Were you allowed to have someone you wanted (from outside of staff at the facility, such as family or friends) to stay with you during labor? | 0, 0 No, never \|  1, 1 Yes, a few times \|  2, 2 Yes, most of the time \|  3, 3 Yes, all the time \|  4, 4 I did not want someone to stay with me |  |
| 170 | Were you allowed to have someone you wanted to stay with you during delivery? | 0, 0 No, never \|  1, 1 Yes, a few times \|  2, 2 Yes, most of the time \|  3, 3 Yes, all the time \|  4, 4 I did not want someone to stay with me |  |
| 245 | Is it acceptable all the time, some of the time, or never to not be allowed to have someone you want stay with you during labor, delivery, or after delivery? | 1, 1 Unacceptable in all instances \| 2, 2 Acceptable in certain instances \| 3, 3 Acceptable in all instances |  |
| 298 | Did anyone accompany you from home to the place  you delivered? | 0, 0 No \|  1, 1 Yes \| | skip to 303 if no |
| 299 | Who accompanied you? | 1, 1 Partner-Husband \| 2, 2 Mother in Law \| 3, 3 Mother \| 4, 4 Sister/Sister in law \| 5, 5 Friend- Neighbour \| 6, 6 Community Health Volunteer \| 7,7 Nurse/Midwife \| 8, 8 Doctor \| 9, 9 Other (specify)___________ |  |
| 300 | if other specify |  |  |
| 301 | Did the person(s) who accompanied you from home stay with you during labor? | 0, 0 No \| 1, 1 Yes \|8, 8 Don't know |  |
| 302 | Did you want this person to stay with you during labor? | 0, 0 No \|  1, 1 Yes \| 8, 8 Don't know |  |
| 303 | If you were to have another baby, would you like someone apart from the person who will deliver your baby to be in the same room as you during your labor? | 0, 0 No \|  1, 1 Yes \| 8, 8 Don't know |  |
| 304 | Who would you want to be in the same room with you during labor?  (Select all that apply) | 1, 1 Partner-Husband \| 2, 2 Mother in Law \| 3, 3 Mother \| 4, 4 Sister/Sister in law \| 5, 5 Friend- Neighbour \| 6, 6 Community Health Volunteer \| 7,7 Nurse/Midwife \| 8, 8 Doctor \| 9, 9 Other (specify)___________ | skip if no |
| 305 | if other specify |  |  |
| 306 | Did the person(s) who accompanied you from home stay with you during the delivery--i.e at the time the baby was being delivered? | 0, 0 No \|  1, 1 Yes \| 8, 8 Don't know | skip if no one accompanied them |
| 307 | Did you want this person to stay with you during the delivery ? | 0, 0 No \|  1, 1 Yes \| 8, 8 Don't know | skip if no one accompanied them |
| 308 | If you were to have another baby, would you like someone apart from the person delivering your baby to be in the same room as you when the baby is being delivered? | 0, 0 No \|  1, 1 Yes \| 8, 8 Don't know |  |
| 309 | Who would you want to be in the same room with you when the baby is being delivered? | 1, 1 Partner-Husband \| 2, 2 Mother in Law \| 3, 3 Mother \| 4, 4 Sister/Sister in law \| 5, 5 Friend- Neighbour \| 6, 6 Community Health Volunteer \| 7,7 Nurse/Midwife \| 8, 8 Doctor \| 9, 9 Other (specify)___________ | skip if no |
| 310 | if other specify |  |  |
| 311 | Did the person(s) who accompanied you from home stay with you after the baby was delivered? | 0, 0 No \|  1, 1 Yes \| 8, 8 Don't know | skip if no one accompanied them |
| 312 | Did you want this person to stay with you after the delivery ? | 0, 0 No \|  1, 1 Yes \| 8, 8 Don't know | skip if no one accompanied them |
| 313 | If you were to have another baby, would you like someone to stay in the same room with you after delivery? | 0, 0 No \|  1, 1 Yes \| 8, 8 Don't know |  |
| 314 | Who would you want to be in the same room with you after delivery? | 1, 1 Partner-Husband \| 2, 2 Mother in Law \| 3, 3 Mother \| 4, 4 Sister/Sister in law \| 5, 5 Friend- Neighbour \| 6, 6 Community Health Volunteer \| 7,7 Nurse/Midwife \| 8, 8 Doctor \| 9, 9 Other (specify)___________ | skip if no |
| 315 | if other specify |  |  |

Guide for Focus Group Discussions

| Main question | Specific prompts | General prompts | Notes |
| --- | --- | --- | --- |
| **Tell us about your birth experience in the health facility?** | Were your relatives allowed to stay with you during labor and or the delivery? How did you feel about that? | Did anyone have similar or different experience?  **Can you tell me more about that?**   1. Why do you say that? 2. What actually happened? 3. Can you walk me through what happened, step by step? 4. Can you give me an example 5. How did that make you feel?   **Why do you think that happened?** |  |

| **Provider Interview Guide** | |  |
| --- | --- | --- |
|  | English |  |
| Question Number (surveys only) | Field Label | Choices |
| 96 | Are women allowed to have someone they want to (from outside of staff at the facility, such as family or friends) stay with them during labor? | 0, 0 No, never \| 1, 1 Yes, a few times \| 2, 2 Yes, most of the time \| 3, 3 Yes, all the time \| 8, 8 Don't know |
| 97 | PROBE ON NO: Why might a woman not be allowed to have someone they want around during their labor?   PROBE ALL: What factors make it difficult for women to have someone stay with them? |  |
| 98 | Are women allowed to have someone they want to stay with them during delivery?  (note, the first was during labor, this is during delivery) | 0, 0 No, never \| 1, 1 Yes, a few times \| 2, 2 Yes, most of the time \| 3, 3 Yes, all the time \| 8, 8 Don't know |
| 99 | PROBE ALL: Why might a woman not be allowed to have someone they want around during their delivery? |  |
|  |  |  |
