# Supplementary material for: Companionship during facility-based childbirth: results from a mixed-methods study with recently delivered women and providers in Kenya
Source: BMC Pregnancy Childbirth. 2018 May 10;18:150. doi: 10.1186/s12884-018-1806-1 (PMC5946503; doi:10.1186/s12884-018-1806-1)
Supplement: Supplementary file 2 — Coding example. (DOCX 96 kb) [file 12884_2018_1806_MOESM2_ESM.docx]

| **Additional file 2: Worked example of coding leading to theme on mistrust of companions as reason for not allowing birth companionship** | | | |
| --- | --- | --- | --- |
| Text | Open codes | Categories (sub-themes) | Theme |
| “like here they like coming with the herbs, since the nurse is one, you leave the mother with the care taker and by the time you are back, you find that they have place for the mother the herb, so it depends on who has brought the client.” | come with herbs; give woman herbs when nurse is not present; depends on type of companion | Fear companions will practice things usually done in home deliveries.  Distrust of companions particularly older mothers-in-law | Distrust of companions |
| “Sometimes you find the mother in second stage, a mother comes in, there are certain things they usually practice home deliveries, so you can find that they start practicing that so we don’t allow that in health care so this is why. Mothers at home give fundal [pressure] and they can touch anywhere with bare hands, yeah so in a normal delivery procedure they remain outside the delivery room. | practicing things done at home like fundal pressure, touching woman with bare hands; don't allow these practices |  |  |
| “Mostly those old mothers, the grannies, the mother in laws who are old. They believe that once a mother comes to the labor ward to deliver, maybe to get a baby as fast as possible, so once they are around, they can discuss and give the mother some medicine to fasten delivery so [because of] that most of us who are midwives are very keen and we don’t want those mothers to come so many of them, they can give herbal medicine or they boil tea concentrated without sugar as they believe that this one will hasten the delivery. That act most of us are not ready to entertain.” | old mothers- in-laws want to hasten labor; give mothers medicine to hasten labor; midwifes alert to having mothers around; giving herbal medicine or concentrated tea without sugar to hasten labor; not ready to entertain such practices |  |  |
| “…some care takers are so stubborn they talk to the nurses as if they know everything that the nurse should be doing.” | care takers stubborn; talk to nurses as if they know everything | Fear of companions being disrespectful to providers and not taking instructions |  |
| “Sometimes, some people want to be the nurse and so they give the nurse orders in there. So such people are told to wait in the labor room. | want to be nurses; order nurses about |  |  |
| “…also it depends on the understanding as some of them will come and don’t want to listen to the instructions of the provider.” | don't want to listen to instructions |  |  |
