# Supplementary material for: Companionship during facility-based childbirth: results from a mixed-methods study with recently delivered women and providers in Kenya
Source: BMC Pregnancy Childbirth. 2018 May 10;18:150. doi: 10.1186/s12884-018-1806-1 (PMC5946503; doi:10.1186/s12884-018-1806-1)
Supplement: Supplementary file 3 — COREQ checklist. (PDF 66 kb) [file 12884_2018_1806_MOESM3_ESM.pdf]

Additional file 3: Consolidated criteria for reporting qualitative studies (COREQ): 32-item checklist

No Item	Guide questions/description	Response	manuscript section
Domain 1: Research team and reflexivity			
Personal Characteristics			
1. Interviewer/facilitator	Which author/s conducted the interview or focus group?	First author trained research assistants to conduct interviews and FGD	Methods: data collection paragraphs 3 and 4
2. Credentials	What were the researcher's credentials? E.g. PhD, MD	Lead Author: MBCHB, MPH, PhD. Research assistants have college degrees, one has a nursing degree	Cover page; methods: data collection paragraphs 3 and 4
3. Occupation	What was their occupation at the time of the study?	Lead author was Postdoctoral fellow at UCSF	N/A
4. Gender	Was the researcher male or female?	All authors and research assistants are female	Methods: data collection paragraphs 3
5. Experience and training	What experience or training did the researcher have?	Taken qualitative methods courses and has previous qualitative research experience	N/A
Relationship with participants			
6. Relationship established	Was a relationship established prior to study commencement?	No	N/A
7. Participant knowledge of the interviewer	What did the participants know about the researcher? e.g. personal goals, reasons for doing the research	Participants told purpose of the research. They did not know the researcher	N/A
8. Interviewer characteristics	What characteristics were reported about the interviewer/facilitator? e.g. Bias, assumptions, reasons and interests in the research topic	Training	Methods: data collection paragraphs 3
Domain 2: study design			
Theoretical framework			

9. Methodological orientation and Theory	What methodological orientation was stated to underpin the study? e.g. grounded theory, discourse analysis, ethnography, phenomenology, content analysis	General qualitative methods in a mixed methods study. We applied grounded theory approaches in the coding, but will not say this was a grounded theory study	N/A
Participant selection			
10. Sampling	How were participants selected? e.g. purposive, convenience, consecutive, snowball	Purposive	Methods: data collection paragraphs 3 and 4
11. Method of approach	How were participants approached? e.g. face-to-face, telephone, mail, email	face to face	Methods: data collection paragraphs 3 and 4
12. Sample size	How many participants were in the study?	8 FGDs with 58 women and 49 interviews with providers	Methods: data collection paragraphs 3 and 4
13. Non-participation	How many people refused to participate or dropped out? Reasons?	None	N/A
Setting			
14. Setting of data collection	Where was the data collected? e.g. home, clinic, workplace	community space and clinic	Methods: data collection paragraphs 3 and 4
15. Presence of non-participants	Was anyone else present besides the participants and researchers?	No	Methods: data collection paragraphs 3 and 4
16. Description of sample	What are the important characteristics of the sample? e.g. demographic data, date	Interview period and demographic information provided	methods: data collection paragraphs 3 and 4; Table 1 and 5
Data collection			
17. Interview guide	Were questions, prompts, guides provided by the authors? Was it pilot tested?	Yes	Supplental file 1
18. Repeat interviews	Were repeat interviews carried out? If yes, how many?	No	N/A

19. Audio/visual recording	Did the research use audio or visual recording to collect the data?	Yes; audio	methods: data collection paragraphs 3 and 4
20. Field notes	Were field notes made during and/or after the interview or focus group?	Yes	methods: data collection paragraphs 3 and 4
21. Duration	What was the duration of the interviews or focus group?	90 mins for FGD and an hour for interviews	methods: data collection paragraphs 3 and 4
22. Data saturation	Was data saturation discussed?	Yes	methods: data analysis paragraphs 2
23. Transcripts returned	Were transcripts returned to participants for comment and/or correction?	No	N/A
Domain 3: analysis and findings			
Data analysis			
24. Number of data coders	How many data coders coded the data?	2	methods: data analysis paragraphs 2
25. Description of the coding tree	Did authors provide a description of the coding tree?	Yes	methods: data analysis paragraphs 2
26. Derivation of themes	Were themes identified in advance or derived from the data?	derived from data	methods: data analysis paragraphs 2
27. Software	What software, if applicable, was used to manage the data?	atlas ti	methods: data analysis paragraphs 2
28. Participant checking	Did participants provide feedback on the findings?	No	N/A
Reporting			
29. Quotations presented	Were participant quotations presented to illustrate the themes / findings? Was each quotation identified? e.g. participant number	Yes	Results: qualitative section from paragraph 12
30. Data and findings consistent	Was there consistency between the data presented and the findings?	Yes	Results: qualitative section from paragraph 12
31. Clarity of major themes	Were major themes clearly presented in the findings?	Yes	Results: qualitative section from paragraph 12

32. Clarity of minor themes	Is there a description of diverse cases or discussion of minor themes?	Yes; results	Results: qualitative section from paragraph 12
