# Supplementary material for: Companionship during facility-based childbirth: results from a mixed-methods study with recently delivered women and providers in Kenya
Source: BMC Pregnancy Childbirth. 2018 May 10;18:150. doi: 10.1186/s12884-018-1806-1 (PMC5946503; doi:10.1186/s12884-018-1806-1)
Supplement: Supplementary file 4 — Multivariate regression of desire for labor companionship and male companions on potential predictors. (DOCX 105 kb) [file 12884_2018_1806_MOESM4_ESM.docx]

| **Additional file 4: Multivariate regression of desire for labor companionship and preference for male partner on potential predictors, PQCC study 2016** | | | | | | | |
| --- | --- | --- | --- | --- | --- | --- | --- |
|  | *Desire labor companion in future* | | |  | *Desire labor companionship from partner in future* | | |
|  | OR | 95% CI | |  | OR | 95% CI | |
| Age |  |  |  |  |  |  |  |
| 15 to 19 years | 1 | [1 | 1] |  | 1 | [1 | 1] |
| 20 to 29 years | 0.84 | [0.51 | 1.40] |  | 0.85 | [0.47 | 1.56] |
| 30 to 48 years | 1.21 | [0.62 | 2.35] |  | 0.68 | [0.32 | 1.46] |
| Currently married | 0.9 | [0.56 | 1.44] |  | 2.56** | [1.45 | 4.50] |
| Number of births |  |  |  |  |  |  |  |
| 1.0 | 1 | [1 | 1] |  | 1 | [1 | 1] |
| 2.0 | 1.56 | [0.70 | 3.44] |  | 1.42 | [0.60 | 3.37] |
| 3.0 | 1 | [0.43 | 2.34] |  | 1.14 | [0.45 | 2.89] |
| 4 or more | 0.84 | [0.36 | 1.99] |  | 1.17 | [0.45 | 3.02] |
| Education |  |  |  |  |  |  |  |
| No school/Primary | 1 | [1 | 1] |  | 1 | [1 | 1] |
| Post-primary/vocational/Secondary | 1.15 | [0.77 | 1.70] |  | 0.92 | [0.58 | 1.45] |
| College or above | 0.58 | [0.31 | 1.06] |  | 2.06* | [1.10 | 3.86] |
| Literacy |  |  |  |  |  |  |  |
| No, cannot write | 1 | [1 | 1] |  | 1 | [1 | 1] |
| Yes, but with some difficulty | 0.83 | [0.32 | 2.13] |  | 0.62 | [0.24 | 1.63] |
| Yes, very well | 0.6 | [0.24 | 1.49] |  | 0.38* | [0.15 | 0.97] |
| Household wealth |  |  |  |  |  |  |  |
| Poorest/poorer | 1 | [1 | 1] |  | 1 | [1 | 1] |
| Middle | 0.92 | [0.58 | 1.45] |  | 1.68 | [1.00 | 2.82] |
| Richer/richest | 0.96 | [0.64 | 1.44] |  | 1.74* | [1.09 | 2.80] |
| Employed | 2.49*** | [1.62 | 3.84] |  | 1.27 | [0.83 | 1.92] |
| Past health facility delivery | 0.87 | [0.44 | 1.73] |  | 1.29 | [0.61 | 2.71] |
| 4plus ANC visits | 1.01 | [0.72 | 1.41] |  | 1.49 | [1.00 | 2.22] |
| Birth Complication | 1.29 | [0.85 | 1.96] |  | 1.15 | [0.72 | 1.85] |
| Tribe |  |  |  |  |  |  |  |
| Luo | 1 | [1 | 1] |  | 1 | [1 | 1] |
| Kuria | 1.45 | [0.94 | 2.24] |  | 0.73 | [0.45 | 1.19] |
| Other | 1.37 | [0.81 | 2.31] |  | 0.97 | [0.54 | 1.74] |
| Religion |  |  |  |  |  |  |  |
| Catholic | 1 | [1 | 1] |  | 1 | [1 | 1] |
| Protestant/Pentecostal | 1.01 | [0.65 | 1.57] |  | 0.58* | [0.34 | 0.99] |
| Seventh Day Adventist | 1.47 | [0.97 | 2.22] |  | 1.03 | [0.66 | 1.60] |
| Other Christian | 0.92 | [0.58 | 1.45] |  | 0.92 | [0.54 | 1.55] |
| Muslim/other religion | 0.42 | [0.13 | 1.36] |  | 0.55 | [0.11 | 2.65] |
| Delivery facility type |  |  |  |  |  |  |  |
| Gov't Hospital | 1 | [1 | 1] |  | 1 | [1 | 1] |
| Gov't HC/Disp | 1.34 | [0.94 | 1.92] |  | 1.52* | [1.02 | 2.26] |
| Mission/Private facility | 1 | [0.61 | 1.66] |  | 1.29 | [0.74 | 2.24] |
| Delivery provider |  |  |  |  |  |  |  |
| Nurse/Midwife | 1 | [1 | 1] |  | 1 | [1 | 1] |
| Doctor/Clinical Officer | 0.8 | [0.49 | 1.30] |  | 0.71 | [0.41 | 1.25] |
| Non-skilled attendant | 0.95 | [0.34 | 2.62] |  | 1.15 | [0.39 | 3.46] |
| 1plus skilled providers | 0.74 | [0.36 | 1.54] |  | 0.84 | [0.37 | 1.89] |
| Delivery Provider sex |  |  |  |  |  |  |  |
| Male | 1 | [1 | 1] |  | 1 | [1 | 1] |
| Female | 0.94 | [0.64 | 1.38] |  | 0.74 | [0.49 | 1.13] |
| Both | 1 | [0.39 | 2.57] |  | 1.71 | [0.65 | 4.48] |
| Accompanied by in (last delivery) |  |  |  |  |  |  |  |
| Partner | 1.03 | [0.71 | 1.48] |  |  |  |  |
| Mother-in-law | 1.42 | [0.96 | 2.10] |  |  |  |  |
| Mother | 1.64 | [0.92 | 2.90] |  |  |  |  |
| Sister/Sister in law | 1.60* | [1.05 | 2.44] |  |  |  |  |
| Friend- Neighbor | 0.9 | [0.58 | 1.41] |  |  |  |  |
| Facility crowded | 0.86 | [0.62 | 1.21] |  |  |  |  |
| Allowed continuous labor support | 2.04*** | [1.46 | 2.87] |  |  |  |  |
| Interviews in community (ref=HF) | 0.8 | [0.57 | 1.10] |  | 0.99 | [0.69 | 1.44] |
| postpartum length => 1 week (ref=<1 | 0.62 | [0.34 | 1.15] |  | 1.68 | [0.81 | 3.52] |
|  |  |  |  |  |  |  |  |
| Constant | 2.28 | [0.64 | 8.11] |  | 0.089*** | [0.022 | 0.36] |
| N | 764 |  |  |  | 872 |  |  |
| Notes: * p<0.05 ** p<0.01 *** p<0.001 | | | |  |  |  |  |
